# Seasonal wild dance of dual endosymbionts in the pear psyllid *Cacopsylla pyricola* (Hemiptera: Psylloidea)

**DOI:** 10.1038/s41598-023-43130-w

**Published:** 2023-09-25

**Authors:** Liliya Štarhová Serbina, Erika Corretto, Juan Sebastian Enciso Garcia, Michela Berta, Tobia Giovanelli, Jessica Dittmer, Hannes Schuler

**Affiliations:** 1 Faculty of Agricultural, Environmental and Food Sciences, Free University of Bozen-Bolzano, 39100 Bolzano, Italy; 2 Department of Botany and Zoology, Faculty of Science, Masaryk University, 60200 Brno, Czech Republic; 3 UMR 1345, Institut Agro, INRAE, IRHS, SFR Quasav, Université d’Angers, Angers, France; 4 Competence Centre for Plant Health, Free University of Bozen-Bolzano, 39100 Bolzano, Italy

**Keywords:** Microbial communities, Environmental microbiology

## Abstract

Most sap-feeding insects maintain obligate relationships with endosymbiotic bacteria that provide their hosts with essential nutrients. However, knowledge about the dynamics of endosymbiont titers across seasons in natural host populations is scarce. Here, we used quantitative PCR to investigate the seasonal dynamics of the dual endosymbionts “*Candidatus* Carsonella ruddii” and “*Ca.* Psyllophila symbiotica” in a natural population of the pear psyllid *Cacopsylla pyricola* (Hemiptera: Psylloidea: Psyllidae). Psyllid individuals were collected across an entire year, covering both summer and overwintering generations. Immatures harboured the highest titers of both endosymbionts, while the lowest endosymbiont density was observed in males. The density of *Carsonella* remained high and relatively stable across the vegetative period of the pear trees, but significantly dropped during the non-vegetative period, overlapping with *C. pyricola*’s reproductive diapause. In contrast, the titer of *Psyllophila* was consistently higher than *Carsonella*’s and exhibited fluctuations throughout the sampling year, which might be related to host age. Despite a tightly integrated metabolic complementarity between *Carsonella* and *Psyllophila*, our findings highlight differences in their density dynamics throughout the year, that might be linked to their metabolic roles at different life stages of the host.

## Introduction

Symbiotic associations between Eukaryotic and Prokaryotic organisms have had a tremendous impact on the diversification of multicellular organisms, contributing to a great proportion of the planet's biodiversity^1,2^. For instance, endosymbiotic bacteria played a central role in shaping the ecological niches of phytophagous insects by enabling them to feed on a nutritionally unbalanced plant sap diet^3–5^. Bacterial endosymbionts of phytophagous insects are often housed within specialized cells (bacteriocytes) aggregated within special organs (bacteriomes) and provide their hosts with essential nutrients lacking in the plant sap^4,6^. This resulted in the establishment of obligate co-diverging host-symbiont associations, accompanied by drastic reductions in the genome size of the symbiotic bacteria until only core housekeeping genes and biosynthetic pathways for the nutrients required by the insect hosts are retained^7–9^. Many sap-feeding hemipteran lineages, such as sternorrhynchans (aphids, adelgids, psyllids, scales, mealybugs) and auchenorrhynchans (planthoppers, spittlebugs, cicadas), are associated with more than one obligate endosymbiont^9^. In most dual endosymbiotic systems studied to date, the primary endosymbiont supplies the host with the majority of essential amino acids (EAAs), whereas the co-primary endosymbiont complements the genes or pathways that are no longer present in the primary endosymbiont^10–13^.

Although endosymbionts provide important benefits, maintaining them also entails fitness costs for the host. For instance, in aphids the titer of the primary endosymbiont *Buchnera* is negatively correlated with the overall host reproductive rate^14^. This is likely due to metabolic costs involved in endosymbiont maintenance^15^. Hence, optimal regulation of endosymbiont titers by the host is crucial to maintain a delicate balance: endosymbiont titers should be as low as possible to reduce the associated costs for the host but as high as necessary to produce sufficient amounts of nutrients and to ensure vertical transmission to the next generation^16–18^. Furthermore, due to different investments in reproduction, the host’s nutritional requirements may vary across the host’s life cycle and between sexes. In addition, females harbour two endosymbiont populations (i.e., in the bacteriome and ovaries)^19,20^, hence endosymbiont titers may at some point increase in females compared to males.

The density of endosymbionts has indeed been shown to be affected by host age, host and endosymbiont genotype, the insect’s host plant, environmental conditions (e.g. temperature, desiccation) and host requirements^14,21,22^. For instance, the quality of the diet has a significant effect on the density of both obligate and facultative endosymbionts in aphids, suggesting that endosymbiont multiplication is regulated by the insect hosts in response to nutrient availability^23–25^, and might be promoted or suppressed by secondary metabolites in the host-plants^26,27^. Depending on the host’s metabolic needs, the number and function of obligate endosymbionts can be regulated by the host in variable ways for different endosymbionts by providing an excess of host-derived metabolites to one endosymbiont and limiting the supply of required nutrients to another one, thereby restricting its growth^15^. Thus, the density of the aphid primary endosymbiont *Buchnera* increased throughout host ontogeny from embryos to young adults, indicating its important role during the different stages of insect development and reproduction, with a subsequent decrease of its titer during later stages of the aging host^18,28,29^. A similar pattern was found in obligate endosymbionts in several species of mealybugs across generations and life stages^21,30^. However, most studies were conducted under controlled environmental conditions and thus might display a much-reduced variation in the endosymbiont titers compared to natural populations, potentially due to the stabilization of the endosymbiont titer in a constant environment^21,31,32^. This constitutes an important limitation regarding the relevance of these results under natural conditions, reflecting a need for studies investigating the factors influencing endosymbiont density in natural host populations.

The symbiosis between psyllids (Hemiptera: Psylloidea) and their maternally inherited primary endosymbiont “*Candidatus* Carsonella ruddii” (thereafter *Carsonella*) is well characterized^33–35^. In many species, an additional endosymbiont co-occurs with *Carsonella* in the bacteriome^11,13,35^. Depending on the psyllid lineage, *Carsonella* is associated with the co-primary endosymbiont “*Ca.* Profftella armatura" (thereafter *Profftella*) in *Diaphorina* spp.^11,36^ or “*Ca.* Psyllophila symbiotica” (thereafter *Psyllophila*) in several *Cacopsylla* species^13,37,38^. *Carsonella* synthesizes most (in the case of *Cacopsylla* spp.) or all (*Diaphorina* spp.) EAAs for the host, while *Psyllophila* complements the production of the remaining EAAs and both *Psyllophila* and *Profftella* produce vitamins and carotenoids^11,13^. The most comprehensive study on the density of obligate endosymbionts in psyllids showed that the titers of both *Carsonella* and *Profftella* increased throughout the development and with the age of *D. citri*^39,40^. Interestingly, the density of *Profftella* was significantly higher than *Carsonella*’s across all analyzed developmental stages and ages of *D. citri*^39,41^. In contrast, host development and age had no effect on the density of *Carsonella* in the pear psyllid *Cacopsylla pyricola* (Psyllidae) from fifth instar immatures to three-week-old adults reared in the laboratory^42^. The titer of the co-primary endosymbiont (which was recently described as *Psyllophila*^13^) varied between three sampling months in a natural host population, suggesting differences in endosymbiont density dynamics between different generations of *C. pyricola*^43^. However, since the titers of both endosymbionts have not been quantified in the same individuals and throughout an entire year, it remains unknown which factors affect the densities of *Carsonella* and *Psyllophila* across the seasons.

*Cacopsylla pyricola* is the vector of ‘*Ca.* Phytoplasma pyri’ causing Pear Decline disease in pear and peach trees in Europe and North America and thus has a serious impact on agricultural production^44–46^. In Central Europe, this species is multivoltine and spends its entire life cycle on pear trees, producing several summer generations and one morphologically different overwintering generation^47,48^. Summer morphs are small, light-coloured and oviposit on green leaf tissues, whereas overwintering morphs are big, dark-coloured and oviposit on dormant wood and later on new leaves^49^. In Central Europe, the overwintering generation starts at the beginning of autumn with the eggs laid by adults of the last summer generation^47^. According to the available literature, the time of the reproductive diapause of *C. pyricola*, characterized by an absence of psyllid mating and their ovarian development, is partly overlapping with the lifespan of overwintering morphs (late autumn–late winter)^44,47,50^. While adults in March and April still belong to the representatives of the overwintering generation, in early spring they exit from diapause and therefore are referred as to post-diapause individuals. Later in spring, post-diapause adults lay the eggs starting the first summer generation^44,47,50^. In contrast to summer morphs, the individuals from the overwintering generation live very long (September–April) owing to reproductive diapause^48^, whereas summer morphs do not survive the frost thus facing reduced longevity (May–October)^47^.

To assess the dynamics of the endosymbionts across the seasons of an entire year covering multiple reproductive generations of the host, we quantified the titers of the dual endosymbionts *Carsonella* and *Psyllophila* in a naturally occurring population of *C. pyricola*. In the current study, we found contrasting patterns for each endosymbiont and showed for the first time that the density dynamics of these dual endosymbionts are not necessarily synchronized, indicating that their respective importance for the hosts varies throughout the life cycle and seasons, as well as between sexes.

## Results

### Differences in endosymbiont titers during the host life cycle and between sexes

The titers of both *Carsonella* and *Psyllophila* were quantified in 144 individuals (60 male, 60 female, 24 immatures) of *Cacopsylla pyricola* (Table S1) collected in the same pear orchard throughout an entire year from February 2020 to February 2021. Summer morphs were collected from May to October with their immatures present from May to August, whereas overwintering morphs were found from November to April with their immatures being sampled in September.

**Figure 1:**
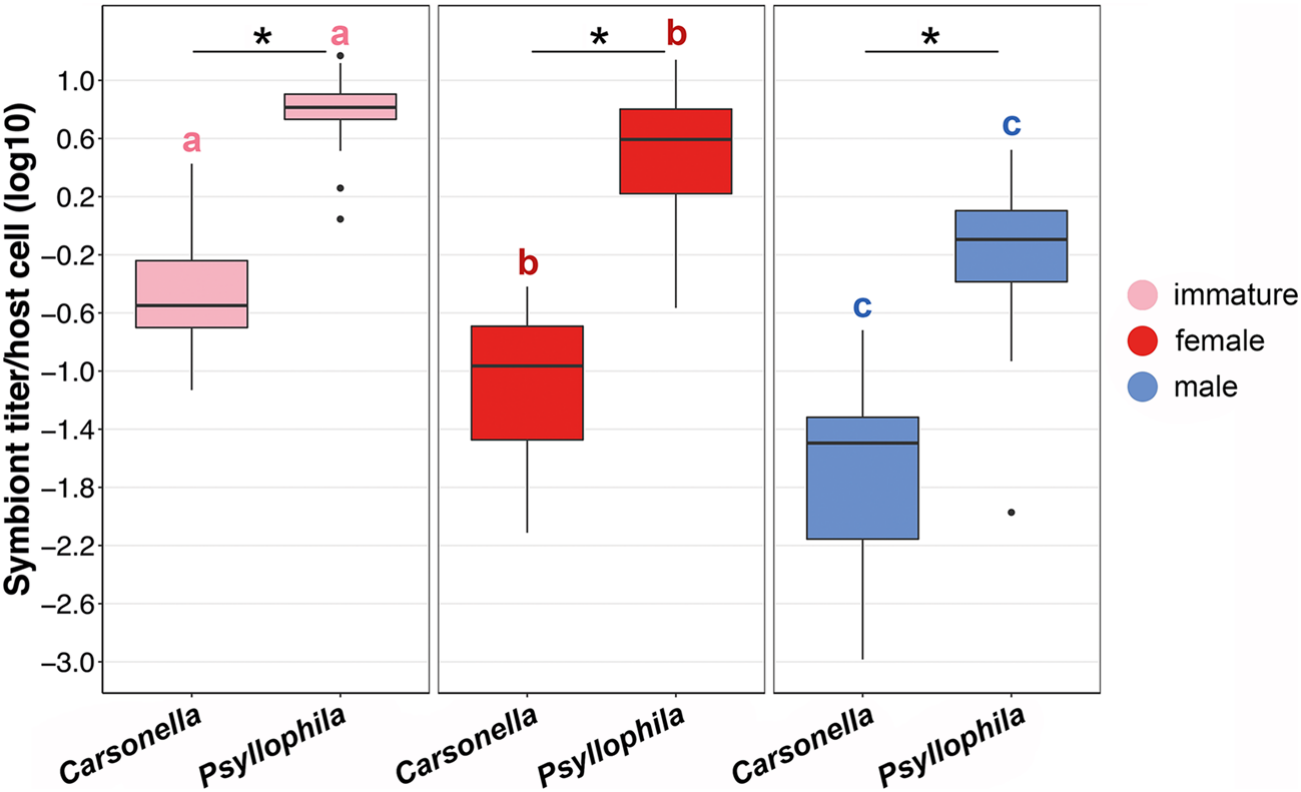
Titers of *Carsonella* and *Psyllophila* based on the developmental stage and sex of the analyzed *C. pyricola* individuals (N = 144). The titer is expressed as endosymbiont titer per host cell. Pink: immatures (N = 24); red: adult females (N = 60); blue: adult males (N = 60). Letters indicate significant differences in the endosymbiont titers between immatures, females and males; asterisks indicate significant differences between *Carsonella* and *Psyllophila* titers for each host life stage and sex.

The density of *Psyllophila* was at least 20 times higher than the density of *Carsonella* across all sampled individuals (mean *Psyllophila* titer/host cell: 3.325 ± 0.281; mean *Carsonella* titer/host cell: 0.149 ± 0.023). This pattern was independent of developmental stage or sex (Wilcoxon rank-sum test: W = 2, *p* < 0.0001 in immatures; W = 8, *p* < 0.0001 in females; W = 50, *p* < 0.0001 in males) (Fig. 1). The titers of both *Carsonella* and *Psyllophila* were found to differ significantly between developmental stages and sexes of *C. pyricola* (Kruskal–Wallis test: χ^2^ = 65.161, df = 2, *p* < 0.0001 for *Carsonella*; χ^2^ = 72.012, df = 2, *p* < 0.0001 for *Psyllophila*) (Fig. 1, S1). Specifically, the density of both *Carsonella* and *Psyllophila* was significantly higher in immature individuals (mean *Carsonella* titer/host cell: 0.477 ± 0.108; mean *Psyllophila* titer/host cell: 6.967 ± 0.721), compared to adults collected in the same time period (from May to September) (mean *Carsonella* titer/host cell: 0.123 ± 0.014; mean *Psyllophila* titer/host cell: 2.281 ± 0.342) (Wilcoxon rank-sum test: W = 1045, *p* < 0.0001 for *Carsonella*; W = 946, *p* < 0.0001 for *Psyllophila*) (Fig. 1). Moreover, the titers of both endosymbionts were significantly higher in females (mean *Carsonella* titer/host cell: 0.129 ± 0.014; mean *Psyllophila* titer/host cell: 4.338 ± 0.411) than in male individuals (mean *Carsonella* titer/host cell: 0.038 ± 0.005; mean *Psyllophila* titer/host cell: 0.937 ± 0.088) during the entire year (Wilcoxon rank-sum test: W = 2082, *p* < 0.0001 in *Carsonella*; W = 3091, *p* < 0.0001 in *Psyllophila*) (Fig. 1).

### Seasonal dynamics of endosymbiont densities across the entire year

1. *Carsonella* density varies between the vegetative and non-vegetative periods of the pear trees

**Figure 2:**
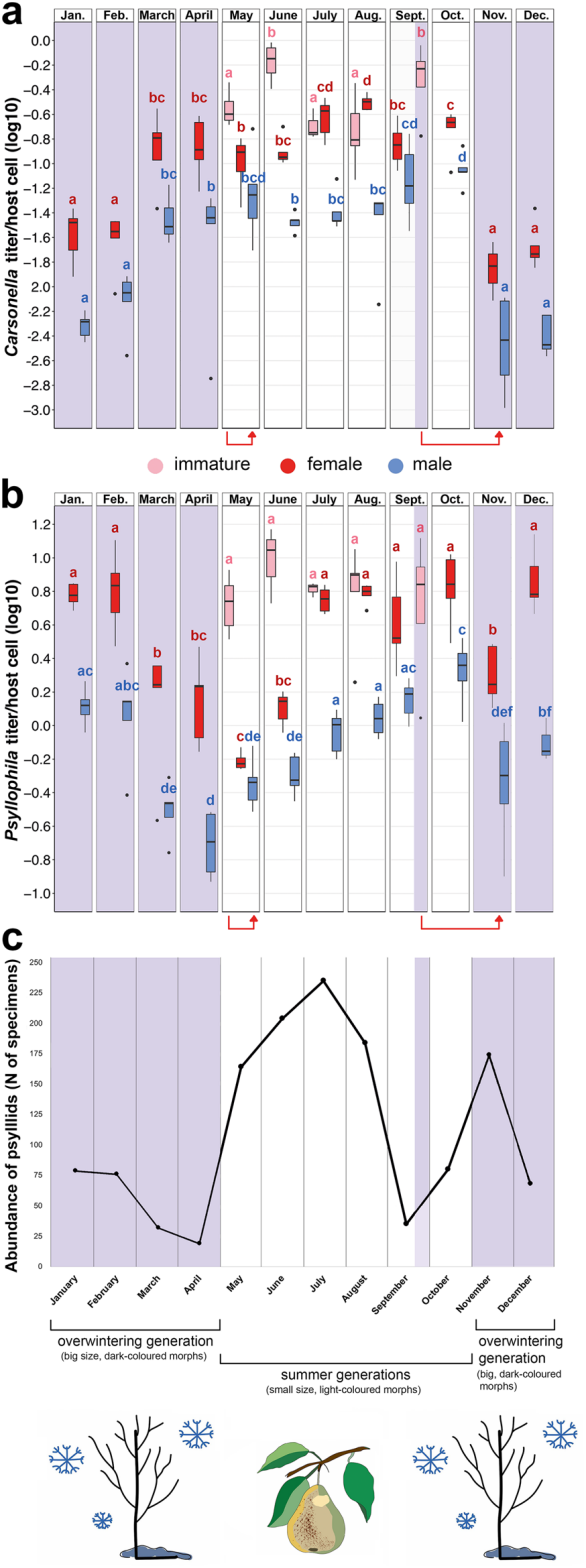
Seasonal dynamics of (**a**) *Carsonella* and (**b**) *Psyllophila* titers across an entire year. The red arrows indicate the transitions between summer and overwintering generations, highlighting the immature stage which will develop into the first adult individuals from the summer and overwintering generations. (**c**) The abundance distribution of adult individuals of *C. pyricola* collected during the sampling year (modified from Štarhová Serbina et al*.*^38^). The shaded areas indicate the period of a single overwintering generation; the remaining areas represent the period of the summer generations. For more details on the analyzed *C. pyricola* individuals, see Table S1.

The titer of *Carsonella* significantly fluctuated along the sampling year (Kruskal–Wallis test: χ^2^ = 79.433, df = 11, *p* < 0.0001 for all months) and ranged from 0.0001 *Carsonella* titer/host cell (in November) to 2.670 (in June) (Fig. 2a; Tables S1 and S2). In immatures, the density of *Carsonella* varied between sampling months (May–September): the highest titers were observed in individuals collected in June and September (mean *Carsonella* titer/host cell: 1.096 ± 0.405 and 0.565 ± 0.153, respectively), which significantly differed from immatures sampled during May, July and August (mean *Carsonella* titer/host cell: 0.247 ± 0.154) (Kruskal–Wallis test with Dunn’s post-hoc June & September vs. May, July & August: *p* ≤ 0.031). In September, representing the start of the overwintering generation, *Carsonella* titers in immatures increased significantly (mean *Carsonella* titer/host cell: 0.565 ± 0.153), compared to the titers of immatures from August (mean *Carsonella* titer/host cell: 0.215 ± 0.065) (Kruskal–Wallis test with Dunn’s post-hoc August vs. September: *p* = 0.031) (Fig. 2a).

**Figure 3:**
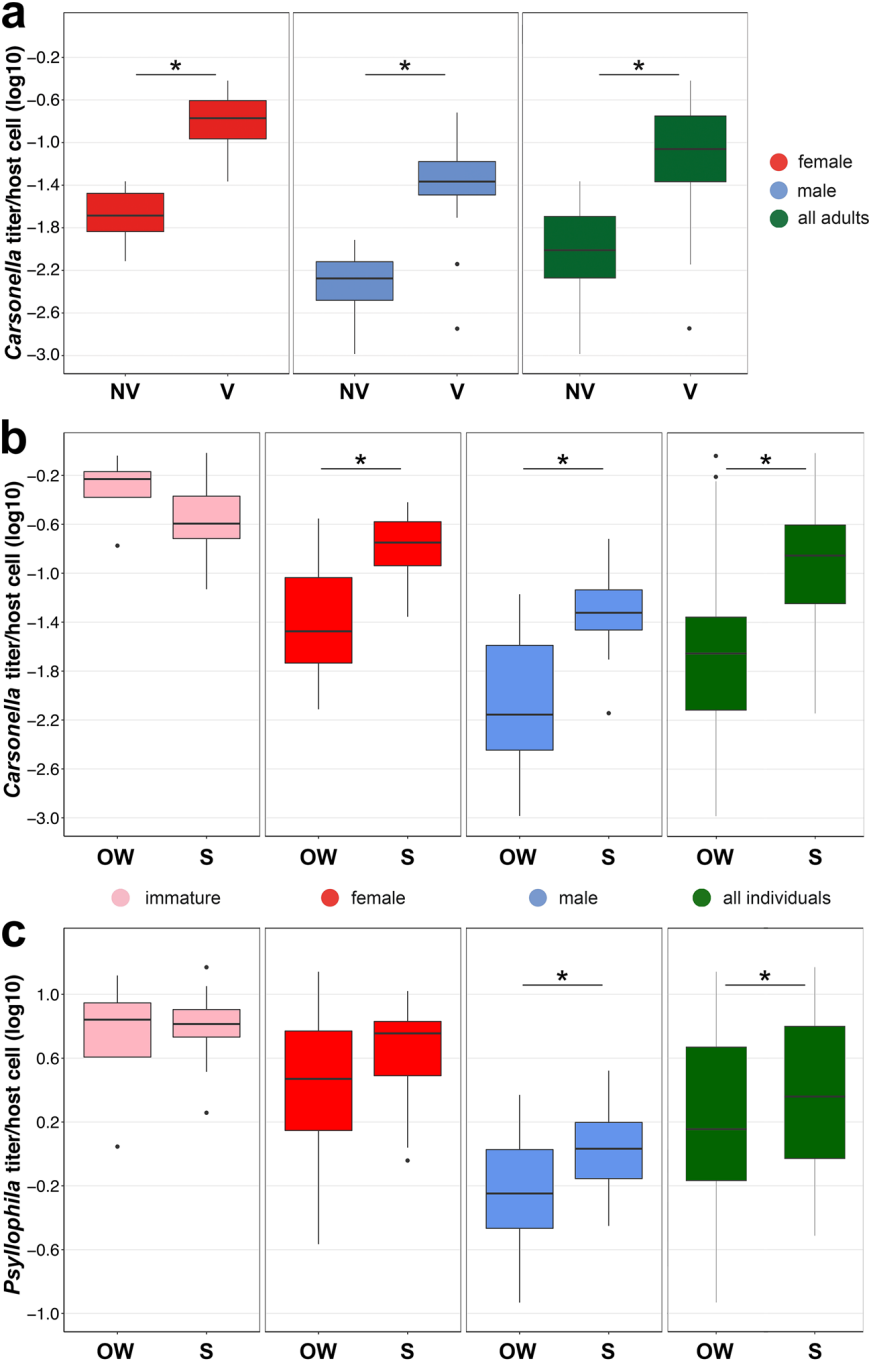
Variation in the titers of (**a**) *Carsonella* during the vegetative (V) and non-vegetative (NV) periods of the pear trees in adult individuals of *Cacopsylla pyricola*. (**b**,**c**) Variation in titers of (**b**) *Carsonella* and (**c**) *Psyllophila* depending on developmental stage and sex of all individuals of *C. pyricola* belonging to overwintering (OW) and summer (S) generations. Asterisks indicate significant differences in the endosymbiont titers between the vegetative and non-vegetative periods of the pear trees and between overwintering and summer generations.

In adults, the density dynamics of *Carsonella* displayed a similar pattern in males and females (Fig. 2a). Overwintering morphs of both sexes collected from November to February (which represent a single generation) showed significantly lower *Carsonella* titers (mean *Carsonella* titer/host cell: from 0.010 ± 0.002 in November to 0.017 ± 0.004 in February), compared to individuals sampled from March to October (mean *Carsonella* titer/host cell: from 0.096 ± 0.027 in March to 0.145 ± 0.158 in October) (Kruskal–Wallis test with Dunn’s post-hoc all months: *p* ≤ 0.048). Among months from March to October, only March and April encompassed the adults from the overwintering generation, while the period from May to October covered the adults from all summer generations (Fig. 2c). This suggests that host age has no effect on the *Carsonella* titer in *C. pyricola*, however, its titer is significantly higher during the vegetative period (March–October), compared to the non-vegetative period (November–February) of the pear trees (Wilcoxon rank-sum test: W = 153, *p* < 0.0001) (Fig. 3a). Since all summer generations fall within the vegetative period, summer morphs of both sexes had significantly higher *Carsonella* titers than overwintering morphs (Wilcoxon rank-sum test: W = 113, *p* < 0.0001 in females; W = 99, *p* < 0.0001 in males) (Fig. 3b).

2. *Psyllophila* density is correlated with host age

Regarding the seasonal dynamics of *Psyllophila*, its titer fluctuated significantly across the sampling year (Kruskal–Wallis test: χ^2^ = 37.416, df = 11, *p* < 0.0001 for all months) ranging from 0.011 *Psyllophila* titer/host cell (in April) to 14.790 (in June) (Fig. 2b; Tables S1 and S3). In immatures, the density of *Psyllophila* remained relatively constant across all five sampling months of the summer generations (May–August) and an overwintering generation (September) (mean *Psyllophila* titer/host cell: 6.967 ± 0.721).

In May, young females from the first summer generation harboured a relatively low amount of *Psyllophila* (mean *Psyllophila* titer/host cell: 0.618 ± 0.035) that increased in June (mean *Psyllophila* titer/host cell: 1.294 ± 0.127). The titer of *Psyllophila* in females then increased significantly between June and July (mean *Psyllophila* titer/host cell in July: 5.694 ± 0.440; Kruskal–Wallis test with Dunn’s post-hoc June vs. July: *p* = 0.0001) and reached its peak in October (mean *Psyllophila* titer/host cell: 7.209 ± 1.345). In the overwintering morphs, the density of *Psyllophila* in female individuals sampled in November (mean *Psyllophila* titer/host cell: 2.123 ± 0.374) was significantly lower than its titers in summer morphs from October (mean *Psyllophila* titer/host cell: 7.209 ± 1.345) (Kruskal–Wallis test with Dunn’s post-hoc October vs. November: *p* = 0.0001). In females from the overwintering generation, the *Psyllophila* titer increased in December compared to November (Kruskal–Wallis test with Dunn’s post-hoc November vs. December: *p* = 0.0001) and remained relatively stable across all overwintering months (December–February) (mean *Psyllophila* titer/host cell: 6.997 ± 0.764) with a value similar to the level of this endosymbiont detected in summer morphs from July to October. Later, the density of *Psyllophila* significantly dropped in senescent post-diapause female individuals from March and April (mean *Psyllophila* titer/host cell: 1.620 ± 0.257) (Kruskal–Wallis test with Dunn’s post-hoc February vs. March and April: *p* = 0.0001) (Fig. 2b).

In males, the seasonal fluctuations in *Psyllophila* titer resembled the variations observed in females, yet with some distinctions. From the beginning of the summer generations, the density of *Psyllophila* in May and June significantly increased, compared to April (Kruskal–Wallis test with Dunn’s post-hoc April vs. May & June: *p* ≤ 0.04), and reached the highest density in October (mean *Psyllophila* titer/host cell: from 0.475 ± 0.078 in May to 2.241 ± 0.384 in October). In November, the *Psyllophila* titer significantly dropped in young male individuals from the overwintering generation (mean *Psyllophila* titer/host cell: 0.563 ± 0.162) (Kruskal–Wallis test with Dunn’s post-hoc October vs. November: *p* = 0.0001), reflecting a pattern similar to that observed in females. In contrast to females, males collected in December harboured only a slightly higher *Psyllophila* density (mean *Psyllophila* titer/host cell: 0.798 ± 0.089), compared to November. Nonetheless, its density significantly increased in older males from the same generation sampled in January (mean *Psyllophila* titer/host cell: from 1.332 ± 0.154 in January) (Kruskal–Wallis test with Dunn’s post-hoc December vs. January: *p* = 0.02) (Fig. 2b) with a significant drop in senescent post-diapause males from the same generation in March and April (mean *Psyllophila* titer/host cell: from 0.328 ± 0.051 in March to 0.173 ± 0.056 in April) (Kruskal–Wallis test with Dunn’s post-hoc January & February vs. March & April: *p* = 0.0001) reaching the lowest titer of *Psyllophila* among males for the entire study period. Similar to *Carsonella*, the density of *Psyllophila* in summer morphs was significantly higher when compared to overwintering morphs (Wilcoxon rank-sum test: W = 1969, *p* = 0.040) (Fig. 3c). However, once senescent post-diapause psyllid individuals from March and April were removed from the analysis, significant differences between the titers of summer and overwintering morphs were no longer observed (Wilcoxon rank-sum test: W = 1677, *p* = 0.929). The latter suggests that seasonal generations of *C. pyricola* have no significant effect on the density of *Psyllophila*.

## Discussion

In the current study, we investigated the density dynamics of the dual endosymbionts *Carsonella* and *Psyllophila* in a natural population of the pear psyllid *Cacopsylla pyricola* (Hemiptera: Psylloidea: Psyllidae) across an entire year and all reproductive generations of the host. Overall, we found consistently higher titers of *Psyllophila* compared to *Carsonella* in all analyzed developmental stages and sexes of *C. pyricola*, while the highest titers of both endosymbionts were harboured by immatures. Our results also demonstrated that the densities of *Carsonella* and *Psyllophila* show striking dissimilarities across the seasons, which might be linked to the differences in the metabolic roles of these endosymbionts.

Our findings on the quantitative predominance of *Psyllophila* over *Carsonella* are in line with the study results on the citrus psyllid *Diaphorina citri*, which also demonstrated higher levels of *Profftella* compared to *Carsonella*^39,41^. A possible explanation for the higher titers of *Psyllophila* and *Profftella*, compared to *Carsonella* in both psyllid species, might be linked to the less efficient transmission mechanism of the co-primary endosymbionts forcing the host to increase the density of these endosymbionts^15,21,51^. Additionally, the association with *Psyllophila* might require fewer metabolic precursors from the host than *Carsonella*, therefore enabling its higher growth. While *Carsonella* produces most essential amino acids (EAAs) for *C. pyricola*, *Psyllophila* complements the genes missing in *Carsonella* for the tryptophan pathway and synthesizes some vitamins and carotenoids^13^. Thus, it is also possible that the nutrients synthesized by *Psyllophila* are required by the psyllid host to a greater extent than the ones provided by *Carsonella*, therefore contributing to the increased population size of *Psyllophila*. Another hypothesis may be linked to the localization of *Psyllophila* in the large syncytial region of the bacteriome, while *Carsonella* colonizes the bacteriocytes surrounding the bacteriome^13^, potentially leading to a smaller overall *Carsonella* population size.

Our work showed that the infection densities of *Carsonella* and *Psyllophila* in *C. pyricola* remained at a relatively low level in male individuals, compared to females. This can be linked to the reduction of endosymbiont titers in males in order to minimize the costs of supporting the endosymbiont populations^14,32,52^. On the other hand, maintaining high endosymbiont titers in females is likely a result of the endosymbiont’s presence not only in the bacteriome to provide the females with nutrients but also in the ovaries to ensure its vertical transmission^19,20^. Our results also indicated that, among all analyzed psyllid individuals, immatures of *C. pyricola* harboured the highest titers of both *Carsonella* and *Psyllophila*, suggesting a high demand for nutrients to support rapid growth during insect development. Yet, since only the last immature instars were included in this study, it is not possible to say during which early stage of host development the endosymbiont titers were at the highest level and thus exactly when the roles of *Carsonella* and *Psyllophila* are the most important for the host. Our observation, however, contradicts the study results from other sap-sucking insect species. For instance, several laboratory studies showed a continuous increase of *Carsonella* in *D. citri*^39^ and of *Buchnera* in the aphid *Acyrthosiphon pisum*^18^ across the host life cycle, reaching a peak of endosymbiont densities in early adults. Also, the titer profiles of both *Carsonella* and *Profftella* in *D. citri*^39^ exhibited a similar growth pattern along different developmental stages of the host. Together, dissimilarities in the results between our research and the study by Dossi et al*.*^39^ could be explained by differences in the psyllid species (*C. pyricola* vs. *D. citri*), endosymbiont genetic features (different strains of *Carsonella*; *Psyllophila* vs. *Profftella*), and/or an effect of the experimental conditions (natural vs. laboratory-reared populations).

Despite the metabolic complementation between *Carsonella* and *Psyllophila*, their density dynamics throughout the sampling year and reproductive generations exhibited strikingly different patterns. Our results suggested that the density of *Carsonella* fluctuated with the psyllid’s reproductive diapause and the non-vegetative period of the pear trees, with similar trends in both males and females. Hence, the titer of *Carsonella* in all analyzed individuals was high and relatively stable throughout the vegetative period of the pear trees (March–October). In contrast to the vegetative period, *Carsonella* density was significantly lower but also relatively stable during the non-vegetative period (November–February). Three hypotheses could explain this density pattern. First, changes in host physiology and behavior during reproductive diapause might contribute to the reduction in *Carsonella*. This may be due to the fact that the insect requires fewer nutrients from *Carsonella* during diapause and therefore reduces the endosymbiont population to lower the metabolic costs of endosymbiont maintenance^42,53^. Second, low winter temperatures might reduce the titer of *Carsonella* by suppressing the endosymbiont proliferation. Third, *Carsonella* titer may vary in response to the phloem composition depending on the vegetative and non-vegetative period of the pear trees. The phloem sap composition during the latter period is affected by cold temperatures which might promote proteolysis, thereby providing sap-feeding insects with increased levels of free amino acids^43,54^, thus reducing the host’s need for *Carsonella*. In fact, it is known that *Carsonella* is responsible for the production of most EAAs for its host^13,34,35^ and all of them, except methionine and tryptophan, can also be found in the pear tree phloem sap^55^. Hence, the host might reduce the *Carsonella* population size due to the lower demand for *Carsonella*-provisioned nutrients that are present in the phloem sap in higher quantities during cold months compared to the remaining part of the year. However, this only applies if the non-migrating psyllid species are feeding on their host-plants during the overwintering stage, which has not yet been determined for *C. pyricola*. The feeding behavior of psyllids during winter was studied only for the plum psyllid *C. pruni*, which is overwintering on conifers, and its ability to feed on them was experimentally demonstrated^56^. Additionally, the seasonal change of secondary metabolites in plants may also influence endosymbiont dynamics by suppressing or promoting the endosymbiont population growth^26,57–59^.

In contrast to *Carsonella*, annual fluctuations of *Psyllophila* densities exhibited a different dynamic, implying no direct effects of seasons or host reproductive diapause on the endosymbiont population. *Psyllophila* titer remained remarkably low in young females and males from the summer generations but increased substantially during the host’s reproductive phase from July to October. The density dynamics of *Psyllophila*, however, differed between males and females: the titer in males increased gradually from May to October, while in females it fluctuated more abruptly with a drastic increase in July but then remained stable for the remainder of the summer generations. Similar to summer morphs, *Psyllophila* density in overwintering morphs first increased with the age of male and female individuals, but this was followed by a decline in the endosymbiont density in senescent post-diapause individuals in March and April. In contrast to the overwintering generation, summer morphs of *C. pyricola* are dying from frost in late autumn^47^, explaining why we could not find similarly senescent summer morphs harbouring a low titer of *Psyllophila*. Therefore, it is likely that *Psyllophila* is not only required to support host development but also plays an important physiological role throughout the adult lifespan. In fact, Le Goff et al*.*^55^ showed that the phloem sap of pear trees is lacking two EAAs (methionine and tryptophan), and *Psyllophila* not only provides vitamins and carotenoids to the host but also complements the genes for tryptophan production that are lost in the *Carsonella* genome^13^. The impact of age on the endosymbiont densities was also observed in several other studies on host-symbiont interactions, indicating that the endosymbiont decrease in aging hosts might be a result of the processes of endosymbiont degradation and autophagy^18,60,61^.

To our knowledge, the current study is the first one measuring the seasonal dynamics of insect dual endosymbionts across various host generations, life stages and sexes, as well as throughout an entire year in a natural environment. We demonstrated that, despite their metabolic complementarity, *Carsonella* and *Psyllophila* display disconnected density dynamics which could be linked to differences in their metabolic roles. These findings show the complex interactions between endosymbionts, their insect hosts and the environment, and highlight the importance of studying the seasonal dynamics of insect endosymbionts under natural conditions.

## Methods

### Psyllid sampling and identification

Adult individuals of *Cacopsylla pyricola* were sampled in the pear orchard Starý Lískovec (Brno) in the Czech Republic across an entire year, from February 2020 to February 2021, using entomological sweep nets and a beating tray. Immatures (4–5th instars) of *C. pyricola* were found from May to September 2020 and collected with a camelhair brush. All individuals were immediately stored in absolute ethanol and kept at − 20 °C. Since it is not possible to differentiate males and females in immature individuals, the terms males and females are used only as a reference to adult psyllid individuals. Adult individuals from summer and overwintering generations were distinguished by their size and colour. Since the *C. pyricola* individuals of this study were sampled in the field, it was not possible to distinguish the individuals from the overlapping summer generations (Fig. 2c). Due to the prolonged longevity of *C. pyricola* from the overwintering generation (September–April)^44,47,50^, we refer to its post-diapause adults (March and April) as senescent individuals. In the case of the summer generations, their morphs face reduced longevity (May–October), associated with frost intolerance and early death in late autumn, before the individuals could have reached the senescent age^44,47,48^. Given this, they are not referred to as senescent individuals in the current study. All the methods were carried out in accordance with relevant Institutional guidelines and regulations.

The sampled adult and immature psyllid specimens were identified based on the morphological keys by Ossiannilsson^62^. The identification of nine immature individuals was additionally confirmed by PCR analysis using the *Cacopsylla*-specific primer set VPm_COI_F2 and VPm_COI_R4 targeting the region tRNACys-tRNATyr-COI^63^. DNA of single immature individuals was extracted using the DNeasy Blood and Tissue Kit (Qiagen) and 25 µl PCR reactions were set up as follows: 2 µl genomic DNA was mixed with 1.75 µl of each primer (at 10 µM), 12.5 µl of DreamTaq PCR Master Mix (2X) (Thermo Scientific) and 7 µl of sterile water. The thermal protocol consisted of an initial denaturation at 95 °C for 3 m; followed by 35 cycles of 95 °C for 30 s, 46 °C for 30 s and 72 °C for 60 s with a final extension step at 72 °C for 10 m. Based on BLAST search, the obtained sequences were identified as *C. pyricola*.

### Quantitative PCR and statistical analyses

In total, we analyzed 144 individuals (60 male, 60 female, 24 immatures) of *C. pyricola*: 10 adult individuals (5 male, 5 female) per month collected over the entire sampling year and 3–7 immature individuals per month collected during the reproductive phase of *C. pyricola* from May to September. Since immatures occur only during five months (May–September), their endosymbiont titers were compared with the adults collected in the same period, excluding the data from the rest of the year (October–April).

The DNA of all samples was extracted using the DNeasy Blood and Tissue Kit (Qiagen). All samples were run in duplicates on a CFX96 real-time PCR system (Bio-Rad, Hercules, CA, USA). Each 10 μl qPCR reaction contained 2 μl of genomic DNA, 5 μl Kapa SYBR qPCR Master Mix 2X (Bio-Rad), 0.25 μl of each primer (10 μM) and 2.5 μl of sterile water. The 16S rRNA genes of *Carsonella* and *Psyllophila* are present in a single copy in the genomes^13^ and were used for quantification. Results were normalized using the single copy host gene wingless (*wg*), as described in Štarhová Serbina et al.^64^. All primers targeting the 16S rRNA gene fragments of *Carsonella* and *Psyllophila* were designed in this study based on the genome sequences published by Dittmer et al*.*^13^. Primers and PCR cycles are summarized in Table 1. The amplification efficiency of the primers for the 16S rRNA and *wg* gene fragments was tested using a standard curve at different annealing temperatures to determine the optimal annealing temperature for the highest amplification efficiency, which ranged from 97.4% to 100.7% for the 16S rRNA fragment of *Carsonella*, from 91 to 118% for the 16S rRNA fragment of *Psyllophila*, and from 89 to 112% for *wg*. To verify the correct amplification of the target PCR fragment across all reactions, a melting curve analysis was performed at the end of each run. Gene copy numbers were determined based on standard curves consisting of 5-point tenfold serial dilutions of longer PCR products of the same genes. A 25 µl volume PCR reaction was set up as follows: 2 µl genomic DNA was mixed with 1.75 µl of each primer (10 µM), 12.5 µl of DreamTaq PCR Master Mix (2X) (Thermo Scientific) and 7 µl of sterile water. The resulting amplicons were purified using AMPure XP beads (Beckman-Coulter) and quantified using the Qubit 1X dsDNA High Sensitivity Assay Kit (Invitrogen).

**Table 1:** Primers and conditions for PCR and qPCR.

Target gene	Primer set	Primer sequence (5'-3')	Amplicon size (bp)	PCR conditions	Reference
16S rRNA	pyr-cars-F-ext pyr-cars-R-ext	GACATCGTTTACTGCATGGACCCACATTGGGACTGAGACAC	463	PCR: 95 °C for 5 m; 35 cycles of 95 °C for 15 s, 57 °C for 30 s and 72 °C for 60 s; 72 °C for 10 m	This study
	pyr-cars-F-int pyr-cars-R-int	CACTGCTACTCCCGAAATTC CAAGCGTTAATCGGAATTATTG	148	qPCR: 95 °C for 3 m; 40 cycles of 92 °C for 30 s, 58 °C for 30 s and 72 °C for 60 s; 72 °C for 10 m	This study
16S rRNA	pyr-psyl-F-ext pyr-psyl-R-ext	CTCAAGGATACAACTTTCAAATTG CTGGAACTGAGACACGGTC	464	PCR: 95 °C for 5 m; 35 cycles of 95 °C for 15 s, 55 °C for 30 s and 72 °C for 60 s; 72 °C for 10 m	This study
	pyr-psyl-F-int pyr-psyl-R-int	GCATTTCACCGCTACACTTG GGGTGCTAGTGTTAATCAG	159	qPCR: 95 °C for 3 m; 40 cycles of 94 °C for 30 s, 56 °C for 30 s and 72 °C for 60 s; 72 °C for 10 m	This study
*Wg*	19F 388R	ACATGYTGGATGAGAYTACCA TCTTGTGTTCTATAACCACGCCCAC	279	PCR: 95 °C for 3 m; 35 cycles of 95 °C for 15 s, 58 °C for 30 s and 72 °C for 60 s; 72 °C for 10 m	^64^
	Wg-202Fq Wg-362Rq	CTCGTCTACCTGGAGACCTC ACGCAGGAAATCACTGTT	186	qPCR: 95 °C for 3 m; 40 cycles of 94 °C for 30 s, 58 °C for 30 s and 72 °C for 60 s; 72 °C for 10 m	^64^

To calculate the endosymbiont titer/host cell ratio, the mean copy number of each endosymbiont was divided by the mean copy number of *wg* for each specimen. All qPCR data were log-transformed and analyzed in R v3.6.3 using the packages agricolae and car. The dataset was tested for normality and homogeneity of variance using Shapiro–Wilk and Levene tests, respectively. Kruskal–Wallis rank-sum test for multiple comparisons was used to analyze the potential differences in *Carsonella* and *Psyllophila* titers between psyllid individuals across different months of the year. Pairwise Wilcoxon rank-sum test was applied to compare the endosymbiont titers between developmental stages and sexes, as well as between summer and overwintering morphs.

### Supplementary Information

Supplementary Information.

## Data Availability

The CO1 sequences generated in this study are available in GenBank under the following accession Numbers, OP899395–OP899403.

## References

[1] Moran NA (2007). Symbiosis as an adaptive process and source of phenotypic complexity. Proc. Natl. Acad. Sci. USA.

[2] Janson EM, Stireman JO, Singer MS, Abbot P (2008). Phytophagous insect-microbe mutualisms and adaptive evolutionary diversification. Evolution.

[3] Dale C, Moran NA (2006). Molecular interactions between bacterial symbionts and their hosts. Cell.

[4] Moran NA, McCutcheon JP, Nakabachi A (2008). Genomics and evolution of heritable bacterial symbionts. Annu. Rev. Genet..

[5] West SA, Fisher RM, Gardner A, Kiers ET (2015). Major evolutionary transitions in individuality. Proc. Natl. Acad. Sci. USA.

[6] Baumann P (2005). Biology of bacteriocyte-associated endosymbionts of plant sap-sucking insects. Annu. Rev. Microbiol..

[7] Gil R, Sabater-Muñoz B, Latorre A, Silva FJ, Moya A (2002). Extreme genome reduction in *Buchnera* spp.: Toward the minimal genome needed for symbiotic life. Proc. Natl. Acad. Sci. USA.

[8] McCutcheon JP, Moran NA (2012). Extreme genome reduction in symbiotic bacteria. Nat. Rev. Microbiol..

[9] McCutcheon JP, Boyd BM, Dale C (2019). The Life of an insect endosymbiont from the cradle to the grave. Curr. Biol..

[10] Mao M, Yang X, Poff K, Bennett G (2017). Comparative genomics of the dual-obligate symbionts from the treehopper, *Entylia carinata* (Hemiptera: Membracidae), provide insight into the origins and evolution of an ancient symbiosis. Gen. Biol. Evol..

[11] Nakabachi A, Piel J, Malenovský I, Hirose Y (2020). Comparative genomics underlines multiple roles of *Profftella*, an obligate symbiont of psyllids: Providing toxins, vitamins, and carotenoids. Gen. Biol. Evol..

[12] Dial DT (2022). Transitional genomes and nutritional role reversals identified for dual symbionts of adelgids (Aphidoidea: Adelgidae). ISME J,.

[13] Dittmer J (2023). Division of labour within psyllids: Metagenomics reveals an ancient dual endosymbiosis with metabolic complementarity in the genus * Cacopsylla*. mSystems, in press.

[14] Chong RA, Moran NA (2016). Intraspecific genetic variation in hosts affects regulation of obligate heritable symbionts. Proc. Natl. Acad. Sci. USA.

[15] Ankrah NYD, Chouaia B, Douglas AE (2018). The cost of metabolic interactions in symbioses between insects and bacteria with reduced genomes. MBio.

[16] Mira A, Moran NA (2002). Estimating population size and transmission bottlenecks in maternally transmitted endosymbiotic bacteria. Microbiol. Ecol..

[17] Hosokawa T, Kikuchi Y, Fukatsu T (2007). How many symbionts are provided by mothers, acquired by offspring, and needed for successful vertical transmission in an obligate insect-bacterium mutualism?. Mol. Ecol..

[18] Simonet P (2016). Direct flow cytometry measurements reveal a fine-tuning of symbiotic cell dynamics according to the host developmental needs in aphid symbiosis. Sci. Rep..

[19] Michalik A (2021). Alternative transmission patterns in independently acquired nutritional cosymbionts of Dictyopharidae planthoppers. MBio.

[20] Vigneron A (2014). Insects recycle endosymbionts when the benefit is over. Curr. Biol..

[21] Parkinson JF, Gobin B, Hughes WOH (2017). The more, the merrier? Obligate symbiont density changes over time under controlled environmental conditions, yet holds no clear fitness consequences: No symbiont density fitness consequences. Physiol. Entomol..

[22] Vogel KJ, Moran NA (2011). Effect of host genotype on symbiont titer in the aphid–*Buchnera* symbiosis. Insects.

[23] Wilkinson TL, Koga R, Fukatsu T (2007). Role of host nutrition in symbiont regulation: impact of dietary nitrogen on proliferation of obligate and facultative bacterial endosymbionts of the pea aphid *Acyrthosiphon pisum*. Appl. Environ. Microbiol..

[24] Chandler SM, Wilkinson TL, Douglas AE (2008). Impact of plant nutrients on the relationship between a herbivorous insect and its symbiotic bacteria. Proc. R. Soc. B..

[25] Snyder AK, McLain C, Rio RVM (2012). The tsetse fly obligate mutualist *Wigglesworthia morsitans* alters gene expression and population density via exogenous nutrient provisioning. Appl. Environ. Microbiol..

[26] Zhang Y-C, Cao W-J, Zhong L-R, Godfray HCJ, Liu X-D (2016). Host plant determines the population size of an obligate symbiont (*Buchnera aphidicola*) in aphids. Appl. Environ. Microbiol..

[27] Guidolin AS, Cônsoli FL (2020). Influence of host plant on oligophagous and polyphagous aphids, and on their obligate symbiont titers. Biologia.

[28] Komaki K, Ishikawa H (2000). Genomic copy number of intracellular bacterial symbionts of aphids varies in response to developmental stage and morph of their host. Insect Biochem. Mol. Biol..

[29] Lu W-N, Chiu M-C, Kuo M-H (2014). Host life stage- and temperature-dependent density of the symbiont *Buchnera aphidicola* in a subtropical pea aphid (*Acyrthosiphon pisum*) population. J. Asia-Pac. Entomol..

[30] Kono M, Koga R, Shimada M, Fukatsu T (2008). Infection dynamics of coexisting Beta- and Gammaproteobacteria in the nested endosymbiotic system of mealybugs. Appl. Environ. Microbiol..

[31] Correa CC, Ballard JWO (2012). *Wolbachia* gonadal density in female and male *Drosophila* vary with laboratory adaptation and respond differently to physiological and environmental challenges. J. Inverteb. Pathol..

[32] Parker BJ, Hrček J, McLean AHC, Brisson JA, Godfray HCJ (2021). Intraspecific variation in symbiont density in an insect–microbe symbiosis. Mol. Ecol..

[33] Thao ML (2000). Cospeciation of psyllids and their primary prokaryotic endosymbionts. Appl. Environ. Microbiol..

[34] Nakabachi A (2006). The 160-Kilobase genome of the bacterial endosymbiont *Carsonella*. Science.

[35] Sloan DB, Moran NA (2012). Genome reduction and co-evolution between the primary and secondary bacterial symbionts of psyllids. Mol. Biol. Evol..

[36] Nakabachi A, Malenovský I, Gjonov I, Hirose Y (2020). 16S rRNA sequencing detected *Profftella*, *Liberibacter*, *Wolbachia*, and *Diplorickettsia* from relatives of the Asian citrus psyllid. Microbiol. Ecol..

[37] Schuler H (2022). Investigating the microbial community of *Cacopsylla* spp. as potential factor in vector competence of phytoplasma. Environ. Microbiol..

[38] Štarhová Serbina L (2022). Microbiome of pear psyllids: A tale about closely related species sharing their endosymbionts. Environ. Microbiol..

[39] Dossi FCA, da Silva EP, Cônsoli FL (2014). Population dynamics and growth rates of endosymbionts during *Diaphorina citri* (Hemiptera, Liviidae) ontogeny. Microbiol. Ecol..

[40] Meng L, Li X, Cheng X, Zhang H (2019). 16S rRNA gene sequencing reveals a shift in the microbiota of *Diaphorina citri* during the psyllid life cycle. Front. Microbiol..

[41] Chu C-C, Gill TA, Hoffmann M, Pelz-Stelinski KS (2016). Inter-population variability of endosymbiont densities in the Asian citrus psyllid (*Diaphorina citri* Kuwayama). Microbiol. Ecol..

[42] Mushegian AA, Tougeron K (2019). Animal-microbe interactions in the context of diapause. Biol. Bull..

[43] Larsson S (1989). Stressful times for the plant stress: Insect performance hypothesis. Oikos.

[44] Horton DR, Higbee BS, Krysan JL (1994). Postdiapause development and mating status of pear psylla (Homoptera: Psyllidae) affected by pear and nonhost species. Ann. Entomol. Soc. Am..

[45] Seemüller E, Schneider B (2004). ‘*Candidatus* Phytoplasma mali’, ‘*Candidatus* Phytoplasma pyri’ and ‘*Candidatus* Phytoplasma prunorum’, the causal agents of apple proliferation, pear decline and European stone fruit yellows, respectively. Int. J. Syst. Evol. Microbiol..

[46] Jarausch, B., Tedeschi, R., Sauvion, N., Gross, J. & Jarausch, W. Psyllid vectors. in *Phytoplasmas: Plant Pathogenic Bacteria*–*II* (eds. Bertaccini, A., Weintraub, P. G., Rao, G. P. & Mori, N.) 53–78 (Springer Singapore, 2019).

[47] Lauterer, P. Results of the investigations on Hemiptera in Moravia, made by the Moravian museum (Psylloidea 2). *Acta Musei Moraviae, Sci. Biol.* **84**, 71–151 (1999).

[48] Hodkinson ID (2009). Life cycle variation and adaptation in jumping plant lice (Insecta: Hemiptera: Psylloidea): A global synthesis. J. Nat. Hist..

[49] Butt BA, Stuart C (1986). Oviposition by summer and winter forms of pear psylla (Homoptera: Psyllidae) on dormant pear budwood. Environ. Entomol..

[50] Horton DR, Guédot C, Landolt PJ (2007). Diapause status of females affects attraction of male pear psylla, *Cacopsylla pyricola*, to volatiles from female-infested pear shoots. Entomol. Exp. Appl..

[51] Campbell MA (2018). Changes in endosymbiont complexity drive host-level compensatory adaptations in cicadas. MBio.

[52] Whittle M, Barreaux AMG, Bonsall MB, Ponton F, English S (2021). Insect-host control of obligate, intracellular symbiont density. Proc. R. Soc. B..

[53] Douglas AE (2000). Reproductive diapause and the bacterial symbiosis in the sycamore aphid *Drepanosiphum platanoidis*: Symbiosis in diapausing aphids. Ecol. Entomol..

[54] Malik NSA, Perez JL, Patt JE, Zibilske LM, Mangan RL (2012). Increased infestation of Asian citrus psyllids on cold treated sour orange seedlings: Its possible relation to biochemical changes in leaves. J. Food Agric. Environ..

[55] Le Goff GJ (2018). What are the nutritional needs of the pear psylla * Cacopsylla pyri*?. Arthrop. Plant Interact..

[56] Gallinger J, Gross J (2018). Unraveling the host plant alternation of *Cacopsylla pruni*—adults but not nymphs can survive on conifers due to phloem/xylem composition. Front. Plant Sci..

[57] Sudha G, Ravishankar GA (2002). Involvement and interaction of various signaling compounds on the plant metabolic events during defense response, resistance to stress factors, formation of secondary metabolites and their molecular aspects. Plant Cell Tissue Organ Cult..

[58] Nishikori K, Morioka K, Kubo T, Morioka M (2009). Age- and morph-dependent activation of the lysosomal system and *Buchnera* degradation in aphid endosymbiosis. J. Insect Physiol..

[59] Koricheva, J. & Barton, K. E. Temporal changes in plant secondary metabolite production: patterns, causes and consequences. in *The Ecol. Plant Second. Metabol.* (eds. Iason, G. R., Dicke, M. & Hartley, S. E.) 34–55 (Cambridge University Press, 2012). 10.1017/CBO9780511675751.004.

[60] Laughton AM, Fan MH, Gerardo NM (2014). The combined effects of bacterial symbionts and aging on life history traits in the pea aphid, Acyrthosiphon pisum. Appl. Environ. Microbiol..

[61] Stoll S, Feldhaar H, Fraunholz MJ, Gross R (2010). Bacteriocyte dynamics during development of a holometabolous insect, the carpenter ant *Camponotus floridanus*. BMC Microbiol..

[62] Ossiannilsson, F. *The Psylloidea (Homoptera) of Fennoscandia and Denmark*. (Brill, E.J., 1992).

[63] Oettl S, Schlink K (2015). Molecular identification of two vector species, *Cacopsylla melanoneura* and *Cacopsylla picta* (Hemiptera: Psyllidae), of Apple Proliferation disease and further common psyllids of Northern Italy. J. Econ. Entomol..

[64] Štarhová Serbina L (2022). *Wolbachia* infection dynamics in a natural population of the pear psyllid *Cacopsylla pyri* (Hemiptera: Psylloidea) across its seasonal generations. Sci. Rep..

