# Supplementary material for: Seasonal wild dance of dual endosymbionts in the pear psyllid Cacopsylla pyricola (Hemiptera: Psylloidea)
Source: Sci Rep. 2023 Sep 25;13:16038. doi: 10.1038/s41598-023-43130-w (PMC10519999; doi:10.1038/s41598-023-43130-w)
Supplement: Supplementary file 1 — Supplementary Information. [file 41598_2023_43130_MOESM1_ESM.pdf]

Seasonal wild dance of dual endosymbionts in the pear psyllid *Cacopsylla pyricola* (Hemiptera: Psylloidea)

Liliya Štarhová Serbina, Erika Corretto, Juan Sebastian Enciso Garcia, Michela Berta, Tobia Giovanelli, Jessica Dittmer & Hannes Schuler

Figure S1. Differences between *Carsonella* and *Psyllophila* titers in **(a)** immature, **(b)** female and **(c)** male individuals of *Cacopsylla pyricola* within the sampling months. All differences are statistically significant with $p = 0.001$.

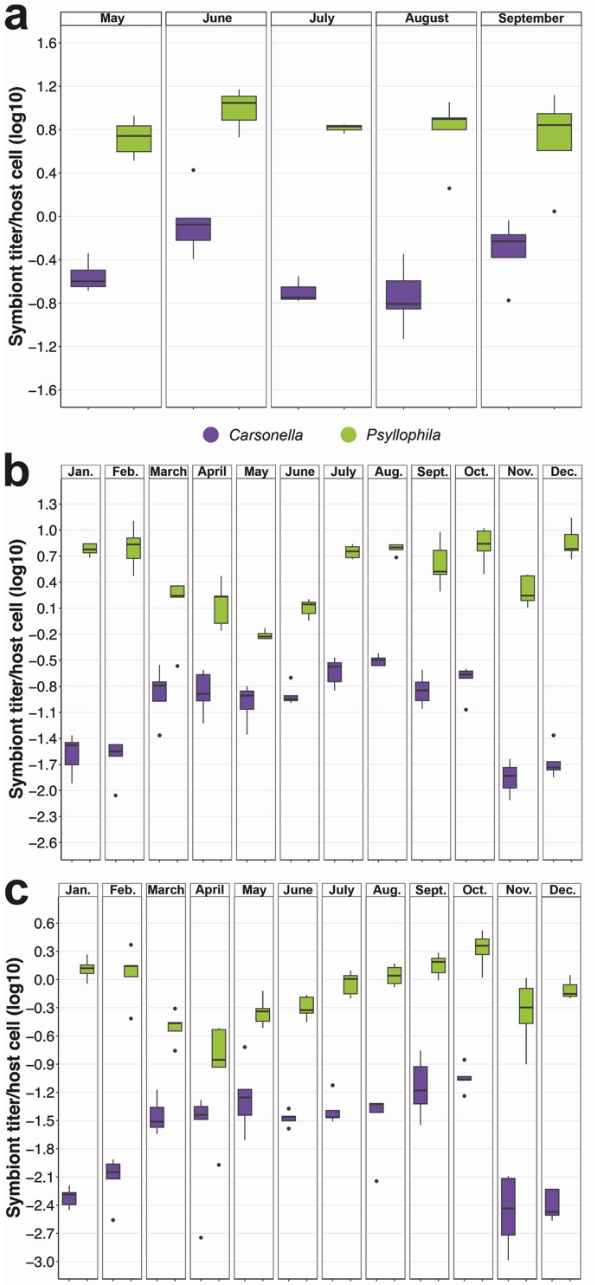

Table S1. The list of *Cacopsylla pyricola* specimens analyzed with qPCR with full information on their developmental stage, sex and collection date. SQ Mean-*Carsonella,* SQ Mean-*Psyllophila* and SQ Mean-*wg* correspond to mean copy numbers of 16S rRNA gene of *Carsonella* and *Psyllophila*, and *wg* gene of the insect host, respectively, based on two technical replicates.

**Specimen ID**	**Sex**	**Developmental stage**	**Collection date**	**SQ Mean-*Carsonella***	**SQ Mean-*Psyllophila***	**SQ Mean-*wg***
jan_f_1	female	adult	17.01.2021	8.30E+04	2.03E+07	4.19E+06
jan_f_2	female	adult	17.01.2021	1.95E+05	3.21E+07	5.87E+06
jan_f_3	female	adult	17.01.2021	5.43E+04	2.69E+07	4.50E+06
jan_f_4	female	adult	17.01.2021	2.01E+05	3.29E+07	4.66E+06
jan_f_5	female	adult	17.01.2021	1.13E+05	2.20E+07	3.16E+06
jan_m_1	male	adult	17.01.2021	7.20E+03	1.60E+06	1.12E+06
jan_m_2	male	adult	17.01.2021	1.87E+04	4.55E+06	3.45E+06
jan_m_3	male	adult	17.01.2021	3.71E+03	1.07E+06	9.21E+05
jan_m_4	male	adult	17.01.2021	2.85E+03	1.48E+06	8.04E+05
jan_m_5	male	adult	17.01.2021	6.34E+03	1.11E+06	1.22E+06
feb_f_1	female	adult	14.02.2021	1.35E+05	6.14E+07	4.81E+06
feb_f_2	female	adult	14.02.2021	1.40E+05	2.66E+07	5.64E+06
feb_f_3	female	adult	14.02.2021	1.74E+05	4.13E+07	5.10E+06
feb_f_4	female	adult	14.02.2021	8.11E+04	1.64E+07	2.40E+06
feb_f_5	female	adult	14.02.2021	2.20E+04	7.46E+06	2.51E+06
feb_m_1	male	adult	14.02.2021	2.58E+04	2.97E+06	2.12E+06
feb_m_2	male	adult	14.02.2021	1.70E+04	2.17E+06	1.56E+06
feb_m_3	male	adult	14.02.2021	2.25E+04	5.90E+06	2.52E+06
feb_m_4	male	adult	14.02.2021	6.56E+03	9.17E+05	2.38E+06
feb_m_5	male	adult	14.02.2021	1.66E+04	2.35E+06	2.19E+06
mar_f_1	female	adult	16.03.2020	1.44E+06	1.36E+07	8.08E+06
mar_f_2	female	adult	16.03.2020	5.23E+05	8.58E+06	4.90E+06
mar_f_3	female	adult	16.03.2020	1.19E+05	7.51E+05	2.76E+06
mar_f_4	female	adult	16.03.2020	4.93E+05	6.93E+06	3.05E+06
mar_f_5	female	adult	16.03.2020	2.45E+06	1.99E+07	8.75E+06
mar_m_1	male	adult	16.03.2020	4.23E+04	5.51E+05	1.58E+06
mar_m_2	male	adult	16.03.2020	3.00E+04	3.35E+05	9.75E+05
mar_m_3	male	adult	16.03.2020	1.01E+05	7.36E+05	1.50E+06
mar_m_4	male	adult	16.03.2020	4.85E+04	1.94E+05	1.11E+06
mar_m_5	male	adult	16.03.2020	1.53E+04	1.90E+05	6.71E+05

apr_f_1	female	adult	13-19.04.2020	7.53E+05	1.01E+07	5.81E+06
apr_f_2	female	adult	13-19.04.2020	2.61E+06	3.16E+07	1.07E+07
apr_f_3	female	adult	13-19.04.2020	2.53E+06	2.00E+07	1.17E+07
apr_f_4	female	adult	13-19.04.2020	1.91E+05	2.72E+06	3.22E+06
apr_f_5	female	adult	13-19.04.2020	5.56E+05	3.60E+06	5.16E+06
apr_m_1	male	adult	13-19.04.2020	1.20E+05	8.21E+05	2.69E+06
apr_m_2	male	adult	13-19.04.2020	2.66E+03	1.58E+04	1.48E+06
apr_m_3	male	adult	13-19.04.2020	1.04E+05	2.33E+05	1.99E+06
apr_m_4	male	adult	13-19.04.2020	5.92E+04	4.78E+05	1.63E+06
apr_m_5	male	adult	13-19.04.2020	6.73E+04	2.90E+05	2.07E+06
may_f_1	female	adult	17.05.2020	2.56E+05	1.64E+06	2.97E+06
may_f_2	female	adult	17.05.2020	1.28E+06	5.42E+06	9.15E+06
may_f_3	female	adult	17.05.2020	1.49E+06	6.92E+06	9.32E+06
may_f_4	female	adult	17.05.2020	2.24E+05	1.16E+06	1.81E+06
may_f_5	female	adult	17.05.2020	1.34E+04	1.71E+05	3.04E+05
may_m_1	male	adult	17.05.2020	7.62E+04	7.63E+05	2.12E+06
may_m_2	male	adult	17.05.2020	5.73E+04	5.06E+05	1.03E+06
may_m_3	male	adult	17.05.2020	1.41E+05	9.54E+05	2.08E+06
may_m_4	male	adult	17.05.2020	2.80E+04	4.36E+05	1.42E+06
may_m_5	male	adult	17.05.2020	8.25E+05	3.27E+06	4.32E+06
jun_f_1	female	adult	14.06.2020	7.32E+05	5.12E+06	3.67E+06
jun_f_2	female	adult	14.06.2020	7.09E+05	5.71E+06	6.29E+06
jun_f_3	female	adult	14.06.2020	1.95E+05	2.52E+06	1.58E+06
jun_f_4	female	adult	14.06.2020	2.09E+05	3.01E+06	2.04E+06
jun_f_5	female	adult	14.06.2020	2.73E+05	2.76E+06	2.52E+06
jun_m_1	male	adult	14.06.2020	5.00E+04	5.10E+05	1.44E+06
jun_m_2	male	adult	14.06.2020	1.19E+05	1.93E+06	2.81E+06
jun_m_3	male	adult	14.06.2020	1.44E+05	1.82E+06	4.14E+06
jun_m_4	male	adult	14.06.2020	3.64E+04	7.53E+05	1.16E+06
jun_m_5	male	adult	14.06.2020	3.04E+04	5.53E+05	1.17E+06
jul_f_1	female	adult	19.07.2020	9.56E+05	1.93E+07	2.80E+06
jul_f_2	female	adult	19.07.2020	4.44E+05	9.65E+06	1.50E+06
jul_f_3	female	adult	19.07.2020	4.31E+05	9.17E+06	1.61E+06
jul_f_4	female	adult	19.07.2020	1.83E+05	6.22E+06	1.29E+06
jul_f_5	female	adult	19.07.2020	2.51E+05	6.48E+06	1.40E+06

jul_m_1	male	adult	19.07.2020	2.66E+04	7.87E+05	7.77E+05
jul_m_2	male	adult	19.07.2020	5.92E+04	1.22E+06	1.73E+06
jul_m_3	male	adult	19.07.2020	3.55E+04	5.87E+05	4.73E+05
jul_m_4	male	adult	19.07.2020	2.94E+04	8.00E+05	7.26E+05
jul_m_5	male	adult	19.07.2020	1.66E+04	3.38E+05	5.36E+05
aug_f_1	female	adult	09.08.2020	1.29E+06	2.14E+07	3.39E+06
aug_f_2	female	adult	09.08.2020	6.24E+05	1.11E+07	2.29E+06
aug_f_3	female	adult	09.08.2020	8.34E+05	2.07E+07	3.03E+06
aug_f_4	female	adult	09.08.2020	8.00E+05	1.61E+07	2.38E+06
aug_f_5	female	adult	09.08.2020	1.10E+06	2.06E+07	3.46E+06
aug_m_1	male	adult	09.08.2020	2.01E+03	1.56E+04	2.80E+05
aug_m_2	male	adult	09.08.2020	6.80E+04	2.09E+06	1.41E+06
aug_m_3	male	adult	09.08.2020	6.70E+04	1.32E+06	1.41E+06
aug_m_4	male	adult	09.08.2020	4.55E+04	9.82E+05	1.18E+06
aug_m_5	male	adult	09.08.2020	1.09E+05	2.94E+06	2.27E+06
sep_f_1	female	adult	27.09.2020	2.29E+05	8.71E+06	2.62E+06
sep_f_2	female	adult	27.09.2020	4.80E+05	2.58E+07	2.71E+06
sep_f_3	female	adult	27.09.2020	1.94E+05	5.53E+06	1.79E+06
sep_f_4	female	adult	27.09.2020	5.63E+05	1.34E+07	2.29E+06
sep_f_5	female	adult	27.09.2020	5.73E+05	7.94E+06	4.04E+06
sep_m_1	male	adult	27.09.2020	2.88E+05	3.75E+06	2.43E+06
sep_m_2	male	adult	27.09.2020	2.43E+04	8.42E+05	8.57E+05
sep_m_3	male	adult	27.09.2020	9.89E+04	2.46E+06	2.08E+06
sep_m_4	male	adult	27.09.2020	8.51E+04	2.17E+06	1.29E+06
sep_m_5	male	adult	27.09.2020	3.18E+05	3.49E+06	1.82E+06
oct_f_1	female	adult	18.10.2020	7.79E+05	2.50E+07	3.59E+06
oct_f_2	female	adult	18.10.2020	5.87E+05	2.59E+07	2.47E+06
oct_f_3	female	adult	18.10.2020	8.47E+05	1.93E+07	3.36E+06
oct_f_4	female	adult	18.10.2020	1.58E+05	5.75E+06	1.85E+06
oct_f_5	female	adult	18.10.2020	5.39E+05	2.67E+07	2.74E+06
oct_m_1	male	adult	18.10.2020	1.30E+05	2.37E+06	2.25E+06
oct_m_2	male	adult	18.10.2020	1.46E+05	2.90E+06	1.57E+06
oct_m_3	male	adult	18.10.2020	1.65E+05	6.38E+06	1.92E+06
oct_m_4	male	adult	18.10.2020	1.77E+05	5.55E+06	2.06E+06
oct_m_5	male	adult	18.10.2020	2.91E+05	4.76E+06	2.08E+06

nov_f_1	female	adult	15.11.2020	2.55E+04	5.10E+06	3.30E+06
nov_f_2	female	adult	15.11.2020	3.30E+04	5.44E+06	3.09E+06
nov_f_3	female	adult	15.11.2020	6.05E+04	9.74E+06	3.28E+06
nov_f_4	female	adult	15.11.2020	3.74E+04	3.23E+06	2.54E+06
nov_f_5	female	adult	15.11.2020	9.55E+04	1.27E+07	4.14E+06
nov_m_1	male	adult	15.11.2020	2.24E+03	3.99E+05	1.17E+06
nov_m_2	male	adult	15.11.2020	1.65E+03	2.00E+05	1.59E+06
nov_m_3	male	adult	15.11.2020	9.14E+03	1.25E+06	2.48E+06
nov_m_4	male	adult	15.11.2020	1.21E+02	1.27E+04	1.58E+04
nov_m_5	male	adult	15.11.2020	1.48E+04	1.89E+06	1.82E+06
dec_f_1	female	adult	13.12.2020	6.10E+04	2.95E+07	3.31E+06
dec_f_2	female	adult	13.12.2020	4.49E+04	1.51E+07	2.60E+06
dec_f_3	female	adult	13.12.2020	1.75E+05	5.61E+07	4.05E+06
dec_f_4	female	adult	13.12.2020	3.08E+04	1.31E+07	2.16E+06
dec_f_5	female	adult	13.12.2020	8.84E+04	1.90E+07	4.11E+06
dec_m_1	male	adult	13.12.2020	5.70E+03	1.47E+06	2.09E+06
dec_m_2	male	adult	13.12.2020	4.27E+03	4.80E+05	7.22E+05
dec_m_3	male	adult	13.12.2020	4.30E+03	8.74E+05	1.38E+06
dec_m_4	male	adult	13.12.2020	8.87E+03	1.68E+06	1.51E+06
dec_m_5	male	adult	13.12.2020	4.22E+03	1.10E+06	1.25E+06
im1	–	immature	17.05.2020	6.11E+05	9.90E+06	1.34E+06
im2	–	immature	17.05.2020	1.63E+06	1.70E+07	4.54E+06
im3	–	immature	17.05.2020	4.55E+05	7.20E+06	2.20E+06
im4	–	immature	17.05.2020	1.23E+06	2.25E+07	5.41E+06
im5	–	immature	24.05.2020	7.91E+05	1.73E+07	3.14E+06
im6	–	immature	24.05.2020	5.54E+05	1.57E+07	2.49E+06
im7	–	immature	24.05.2020	1.30E+06	3.88E+07	4.58E+06
im8	–	immature	14.06.2020	1.65E+06	2.18E+07	1.96E+06
im9	–	immature	14.06.2020	8.86E+05	–	9.21E+05
im10	–	immature	14.06.2020	2.21E+06	–	8.28E+05
im11	–	immature	14.06.2020	4.82E+05	1.76E+07	1.19E+06
im12	–	immature	14.06.2020	8.60E+05	7.67E+06	1.43E+06
im13	–	immature	12.07.2020	3.51E+05	1.48E+07	2.11E+06
im14	–	immature	12.07.2020	4.76E+05	1.80E+07	2.67E+06
im16	–	immature	19.07.2020	3.45E+05	7.16E+06	1.23E+06

im17	–	immature	30.08.2020	1.43E+05	3.50E+06	1.93E+06
im18	–	immature	30.08.2020	4.10E+05	1.30E+07	1.61E+06
im19	–	immature	30.08.2020	8.60E+05	2.15E+07	1.91E+06
im20	–	immature	30.08.2020	2.07E+05	1.17E+07	1.48E+06
im21	–	immature	30.08.2020	1.62E+05	6.55E+06	1.04E+06
im22	–	immature	07.09.2020	1.95E+05	1.29E+06	1.16E+06
im23	–	immature	13.09.2020	4.85E+05	6.96E+06	5.30E+05
im24	–	immature	13.09.2020	1.36E+05	1.72E+06	2.22E+05
im25	–	immature	20.09.2020	8.38E+05	9.21E+06	1.48E+06

Table S2. Effect of sampling months on *Carsonella* titers in immature, female and male individuals of *Cacopsylla pyricola*. Significant differences are represented by *p*-values in bold.

immatures

	May	June	July	August	September
May					
June	**0.0226**				
July	0.3357	**0.0138**			
August	0.2178	**0.0049**	0.8491		
September	0.2178	0.3115	0.0740	**0.0309**	

females

	January	February	March	April	May	June	July	August	September	October	November	December
January												
February	0.8914											
March	**0.0001**	**0.0001**										
April	**0.0002**	**0.0001**	0.9545									
May	**0.0026**	**0.0016**	0.3608	0.3968								
June	**0.0009**	**0.0005**	0.5958	0.6307	0.7351							
July	**0.0001**	**0.0001**	**0.0305**	**0.0259**	**0.0019**	**0.0061**						
August	**0.0001**	**0.0001**	**0.0011**	**0.0009**	**0.0001**	**0.0002**	0.2817					
September	**0.0001**	**0.0001**	0.9700	0.9386	0.3468	0.5784	**0.0326**	**0.0012**				
October	**0.0001**	**0.0001**	0.3396	0.3130	0.0499	0.1202	0.2817	**0.0220**	0.3468			
November	0.2582	0.3261	**0.0001**	**0.0001**	**0.0001**	**0.0001**	**0.0001**	**0.0001**	**0.0001**	**0.0001**		
December	0.6307	0.7527	**0.0001**	**0.0001**	**0.0006**	**0.0002**	**0.0001**	**0.0001**	**0.0001**	**0.0001**	0.5082	

males

	January	February	March	April	May	June	July	August	September	October	November	December
January												
February	0.4664											
March	**0.0026**	**0.0224**										
April	**0.0056**	**0.0463**	0.7956									
May	**0.0001**	**0.0006**	0.1958	0.1124								
June	**0.0039**	**0.0336**	0.8803	0.8913	0.1502							
July	**0.0008**	**0.0063**	0.6971	0.4988	0.4130	0.5890						
August	**0.0007**	**0.0056**	0.6606	0.4664	0.4436	0.9510	0.9510					
September	**0.0001**	**0.0001**	0.0722	**0.0353**	0.6606	**0.0485**	0.1823	0.1958				
October	**0.0001**	**0.0001**	**0.0059**	**0.0027**	0.1657	**0.0039**	**0.0213**	**0.0235**	0.3835			
November	0.8803	0.3835	**0.0017**	**0.0038**	**0.0001**	**0.0026**	**0.0005**	**0.0004**	**0.0001**	**0.0001**		
December	0.7836	0.2966	**0.0011**	**0.0026**	**0.0001**	**0.0017**	**0.0003**	**0.0003**	**0.0001**	**0.0001**	0.8803	

Table S3. Effect of sampling months on *Psyllophila* titers in immature, female and male individuals of *Cacopsylla pyricola*. Significant differences are represented by *p*-values in bold.

immatures

	May	June	July	August	September
May					
June	0.8269				
July	0.8269	0.8269			
August	0.8269	0.8269	0.8269		
September	0.8269	0.8269	0.9238	0.8269	

females

	January	February	March	April	May	June	July	August	September	October	November	December
January												
February	0.9601											
March	**0.0001**	**0.0001**										
April	**0.0001**	**0.0001**	0.8356									
May	**0.0001**	**0.0001**	0.0804	0.1334								
June	**0.0001**	**0.0001**	0.5995	0.7206	0.2914							
July	0.6571	0.6145	**0.0004**	**0.0001**	**0.0001**	**0.0001**						
August	0.9728	0.9472	**0.0001**	**0.0001**	**0.0001**	**0.0001**	0.6723					
September	0.2307	0.2081	**0.0040**	**0.0018**	**0.0001**	**0.0004**	0.5583	0.2398				
October	0.6903	0.7206	**0.0001**	**0.0001**	**0.0001**	**0.0001**	0.3384	0.6876	0.0844			
November	**0.0003**	**0.0002**	0.6903	0.5583	**0.0223**	0.2914	**0.0018**	**0.0003**	**0.0176**	**0.0001**		
December	0.7784	0.8211	**0.0001**	**0.0001**	**0.0001**	**0.0001**	0.4334	0.7635	0.1187	0.9057	**0.0001**	

males

	January	February	March	April	May	June	July	August	September	October	November	December
January												
February	0.6018											
March	**0.0001**	**0.0001**										
April	**0.0001**	**0.0001**	0.3424									
May	**0.0001**	**0.0005**	0.2686	**0.0403**								
June	**0.0002**	**0.0011**	0.1739	**0.0219**	0.8060							
July	0.1041	0.2686	**0.0005**	**0.0001**	**0.0139**	**0.0255**						
August	0.4988	0.8575	**0.0001**	**0.0001**	**0.0016**	**0.0034**	0.3746					
September	0.6946	0.3547	**0.0001**	**0.0001**	**0.0001**	**0.0001**	**0.0427**	0.2966				
October	0.1939	0.0695	**0.0001**	**0.0001**	**0.0001**	**0.0001**	**0.0039**	0.0610	0.3673			
November	**0.0003**	**0.0016**	0.1387	**0.0162**	0.7099	0.8897	**0.0350**	**0.0045**	**0.0001**	**0.0001**		
December	**0.0219**	0.0733	**0.0039**	**0.0002**	0.0695	0.1175	0.4988	0.1320	**0.0074**	**0.0005**	0.1455	
